# Naturalist's Monthly Report

**Published:** 1811-05

**Authors:** 


### NATURALIST'S MONTHLY REPORT.

### MARCH.

Love's pleasing ferment gently now begins

To warm the flowing blood.

No weather could possibly have been more acceptable at this season of the year, than that which we have experienced during the course of the present month. The crops are all looking well. Nor have we yet had any of those furious gales which are usually expected about this time. There were strong gales from west-north-west on the 3d, and from west-south-west on the 6th, 7th, and 8th, and these were the only boisterous days we have had. The wind was westerly or north-west from the 1st to the 5th, in the afternoon of which day it was south. On the 6th, 7th, and 8th, it was west-south-west; on the 9th, north-east, and afterwards west. On the 12th it was easterly, and so continued till the 18th, when it veered round to west. On the 22d it was north; and from the following day to the end of the month easterly.

There was rain on the 1st, 5th, 6th, 7th, 8th, and 22d, but during all the remainder of the month the weather was dry.

March 5th. Rooks have begun to build their nests; and Daffodils are in flower.

The capsules of several species of moss now adorn the banks of hedges and ditches, and the tops of old walls.

March

March 7th. The marsh marygold *(caltha palustris)* is in flower, and give to all the wet meadows a golden hue.

March 8th. Several kinds of insects crawl out of their hybernacula in old buildings, particularly spiders, millepedes, and a species of the glowbeetle, or darkling, (*tenebrio mortisagus* of Linnæus, *blaps mortisaga*, of Marsham).

March 10th. The vernal whitlow-grass (*draba verna*) and purple dead nettle *(lamium purpureum)* are in flower.

March 11th. Some of the smaller kinds of ants are busily employed in opening their holes, and clearing their nests. On attentively observing them, they are seen to bring out grains of sand or other small objects which incommode them in their habitation, and to deposit them at a little distance on the exterior of their holes.

March 12th. A caterpillar was this day seen crawling upon the road. Several dark-coloured butterflies were flitting about the fields.

March 14th. The farina of the male yew tree is blown off by the wind in great quantity.

The plumage of all the small birds is now in the very height of its beauty. Bird-catchers technically call the plumage, at this season, their "wedding garments."

The flowers of some of the willows begin to fade.

March 18th. The leaves of the lilac and weeping-willow appear. Primroses and violets are in flower.

March 20th. Water lizards are seen in abundance in two or three of the shallow and gravelly ponds of this neighbourhood. But I have not yet remarked that they have begun to spawn.

March 21. The roads, which, only a few days ago, had pools of water standing in almost every hollow, are now quite dry and dusty.

March 24. The leaf-buds of the mulberry tree appear nearly ready to burst; but it is probable that these trees will not be in leaf for several days. The leaves of the bramble, woodbine and elder, have been out some time.

March 26th. A species of wood-bug, which I think is *cimex baccarum*, was this day brought to me.

The scentless violet *(viola canina)* and common stitchwort *(stellaria holostea)* are in flower.

March 28th. This was a peculiarly hot day for the season. Insects of numerous kinds were in active employment. Bees were flying about such plants as were in flower; sand wasps (*ammophila vulgaris)* about sandy banks; and *opatrum sabulosum*, several species of *curculio*, and small *carabi*, crawling about among the stunted vegetation of the road sides.

March 30th. Lapwings fly screaming over the wet meadows.

March 31st. The easterly winds which have prevailed for the last nine days of the month have been extremely seasonable. They have tended considerably to check vegetation, which, during the preceding warm weather, was making too rapid a progress for this early part of the year. I have not yet remarked that any of the standard fruit trees are in flower.

*Hampshire.*

